# Bacteriological diagnosis of osteoarticular infections caused by *Kingella kingae*; a narrative review

**DOI:** 10.3389/fped.2024.1520636

**Published:** 2025-01-07

**Authors:** Giacomo De Marco, Oscar Vazquez, Elio Paris, Blaise Cochard, Christina Steiger, Romain Dayer, Dimitri Ceroni

**Affiliations:** ^1^ Pediatric Orthopedic Unit, Pediatric Surgery Service, Geneva University Hospitals, Geneva, Switzerland; ^2^ Faculty of Medicine, University of Geneva, Geneva, Switzerland

**Keywords:** *Kingella kingae*, pediatric, orthopedic, osteoarticular infections, early childhood

## Abstract

In recent years, advancements in modern laboratory diagnostics have identified *Kingella kingae* (*K. kingae*) as the major cause of osteoarticular infections in early childhood. The introduction of novel diagnostic methods has ushered in a new era, transitioning from underrated infections to recognizing *K. kingae* as the primary etiology of skeletal system infections in children. This article provides a new perspective on *K. kingae*, exploring innovative diagnostic methods that have improved and will continue to transform the management of these infections.

## 1 Introduction

Since 1988, Pablo Yagupsky, a Professor of Pediatrics and Clinical Microbiology at the Ben-Gurion University of the Negev, Israel, has been warning the scientific community about the significant role played by *Kingella kingae* (*K. kingae*) in osteoarticular infections. His discovery, which he modestly defined as serendipitous, sparked exponential interest in *K. kingae*, leading to significant advances in its microbiological diagnosis over the last two decades. The number of cases of osteoarticular infections (OAIs) attributed to *K. kingae* has drastically increased, particularly since the 2000s.

*K. kingae* is now regarded as the leading bacterial cause of osteoarticular infections, especially in children under 48 months of age (1–3). In addition to classical acute hematogenous osteomyelitis and septic arthritis, this pathogen can cause atypical osteoarticular infections, including spondylodiscitis (4–10), subacute osteomyelitis (11, 12), pyomyositis (13), bursitis (14), and tendon sheath infections (15–17). Regardless of the infection site, *K. kingae* OAIs are typically characterized by mild clinical presentations and modest inflammatory responses, often resulting in few symptoms evocative of OAI (1–3, 18–20). In view of the poor recognition of OAI due to *K. kingae* and the difficulties in establishing their diagnosis, research over the last 2 decades has focused on ways to improve recognition of these infections. This narrative review tries to summarize more than 20 years of observations of OAI caused by *K. kingae*, focusing specifically on all the strategies that have been developed to establish the bacteriological diagnosis. To do this, we have identified the most pertinent and recent literature on this specific topic using recognized and relevant databases such as Pubmed, Embase and the Cochrane Database of Systematic Reviews. The relevant literature was thereafter analyzed and we provided a critical discussion of it, trying to identify new insights.

## 2 The limitations of gram stain in *K. kingae* diagnosis

Still today, the traditional Gram stain remains of current practice in osteoarticular infections since it combines technical simplicity, inexpensive equipment, and rapid execution time (21). Gram staining is routinely performed on osteoarticular samples like synovial fluid or bone tissue aspirates to confirm infection, suggest the presence of a specific species, and guide the initial antibiotic therapy (21). However, Gram staining has proven ineffective in diagnosing *K. kingae*-related OAIs. In fact, Gram stains are rarely positive in *K. kingae* cases, likely due to the low bacterial concentration in samples (an average of 15 CFUs per ml) (22). Moreover, Gram stains may yield misleading results, as the Gram-negative coccobacilli can be difficult to distinguish from fibrin clumps (22–25). Therefore, when the clinical presentation and biological parameters strongly suggest *K. kingae* OAI, performing a Gram stain may be not recommended, as it is likely to be negative and it will waste part of the precious sample.

## 3 The inadequacy of conventional bacteriological techniques

*K. kingae* is a facultative anaerobic, β-hemolytic, Gram-negative organism that is notoriously difficult to grow on routine solid cultures of blood or body fluids such as bone exudates or synovial fluid (25). Its isolation rate in standard cultures is less than 10% in confirmed cases (9). To overcome this issue, clinical specimens should be inoculated into aerobic blood culture vials (BCV), specifically onto trypticase soy agar with 5% sheep blood, or chocolate agar. The inoculation of a small sample into a large volume of liquid medium dilutes potential inhibitory factors, improving bacterial survival and detection (26).

Thus, a variety of automated or manual blood culture systems, such as BACTEC (Becton Dickinson, Cockeysville, MD, USA), BacT/Alert (OrganonTeknika Corporation, Durham, NC,USA), Isolator 1.5 Microbial Tube (Wampole Laboratories, Cranbury, NJ, USA), or Hemoline DUO (bioMérieux, Lyon, France), were developed for improving the the yield of cultures (26, 27). No controlled study has been performed to identify the best blood culture system for this purpose (21).

Although these techniques have improved the detection of pathogens, a significant number of purulent specimens seeded onto solid media still resulted in negative cultures (25). Consequently, the etiology of invasive diseases caused by *K. kingae*, such as septic arthritis and osteomyelitis, remained undetected in young children, leading probably to their classification as “culture-negative bone infections of unknown origin” (25).

## 4 The advent of nucleic acid amplification assays

Since the early 2000s, nucleic acid amplification assays (NAAAs) have enabled the detection of infinitesimal quantities of bacterial RNA and DNA, offering clinicians powerful tools for identifying bacteriological agents in clinical samples (28, 29). These molecular techniques can detect the pathogen within hours, regardless of the pathogen's viability, or previous antibiotic exposure (21). Initially, NAAAs targeted the 16S rRNA gene, which is present in all bacteria. The prokaryotic 16S rRNA gene is approximately 1,500 bp long, with nine variable regions interspersed between conserved regions (21). Variable regions of the 16S rRNA gene are frequently used for phylogenetic classification of genus or species in diverse microbial populations.

However, the sensitivity of PCR tests targeting the 16S rRNA gene proved insufficient for detecting OAIs caused by *K. kingae* [sensitivity 300 CFUs/ml (30)], since *K. kingae* is often present in very low concentrations in synovial fluid aspirates (on average 15 CFUs/ml) (30, 31).

Subsequent advances in NAAAs have significantly improved detection, with species-specific real-time PCR assays developed to target genes unique to *K. kingae*, including the *rtxA* and the *rtx* B genes that encodes the *RTX* toxin (30), the *groEL* gene (also called *cpn60*) that encodes the chaperonin 60 protein (32), and the *mdh* gene (malate dehydrogenase) (33). A comparative study suggested that PCR test targeting the *mdh* gene is probably more sensitive than those targeting the *groEL* gene and the *rtx* locus, making it the preferable molecular diagnostic assay (33). These molecular methods can also detect antibiotic-resistance-associated genes, rendering traditional cultures methods obsolete and enabling targeted antimicrobial therapy (32). Since its availability, this new diagnostic approach has provided irrefutable evidence that *K. kingae* has become the most common pathogen guilty for primary infections in bones, joints, intervertebral discs, and tendon sheaths, especially among children aged 6–48 months old (1–3, 34).

## 5 Detecting oropharyngeal *K. kingae* carriage as a diagnostic tool

Real-time PCR assays targeting oropharyngeal samples have shown high sensitivity and specificity for detecting *K. kingae* colonization. Since *K. kingae* colonization of the oropharynx is a prerequisite for bloodstream invasion and subsequent musculoskeletal infection, some researchers have proposed non-invasive diagnostic on detecting oropharyngeal carriage (35). The sensitivity and the specificity of the test were 100% and 90.5%, while its accuracy was estimated to be 93% (35). These assays have proven especially useful in diagnosing OAIs in cases where small joints are affected or when sampling is contraindicated, such as during spondylodiscitis (4, 7, 10). However, the predictive value of the test is limited, as the oropharyngeal carriage rate is 10%–12% among the young pediatric population, reducing by the same the predictive value of the NAAT result (21). This notwithstanding, many emergency centers have adopted this diagnostic strategy which reconciles technical simplicity, inexpensive equipment, and rapid execution time.

## 6 The rise of metagenomic next-generation sequencing (mNGS)?

Over the last years, the experience has shown us that even when sensitive NAATs were employed, failure to identify a pathogen could occur in a large fraction of bone and joint infections (1–3), indicating that novel diagnostic methods proved essential for improving the recognition of their bacterial etiology. This problem is even more pronounced for *K. kingae,* because the bacterial load present in the samples can be very low (31). The introduction of next-generation sequencing (NGS) technology has marked a major step forward in identifying microorganisms (28). NGS is a technique that makes possible the fast sequencing of the base pairs in entire genomes or in targeted regions of RNA or DNA samples.

This revolutionary technology, which offers speed, scalability, and ultra-high throughput, enables the rapid sequencing of every genome present in a clinical sample (usually blood, CSF, or other normally sterile body fluids), obtaining millions of DNA strands, and thus reducing the requisite for the traditional cloning methods used in previous genome sequencing techniques (28). In theory, mNGS arbitrarily amplify all the germs present in a clinical sample; it can detect any pathogen, and, about it, this technology can currently unambiguously identify >1,400 species, whereas the turnaround time has been shortened to 1–2 days (36). Interestingly, the detection and identification can be performed without *a priori* knowledge of the suspected etiologic agent, and, since a comprehensive database grounded on single nucleotide polymorphisms is available, a resolution at the subspecies or strain level can be achieved (37). NGS will probably become indispensable in microbiology since it will replace, with its genomic definition of pathogens, the conventional characterization of pathogens by staining properties, study of morphology, and metabolic criteria. The characterization of the pathogens' genomes better will define what they are and will be able to bring crucial information about drug sensitivity. We can expect that NGS will move towards a more focused amplification of specific genomic regions of interest instead of massive simultaneous parallel sequencing as soon as the pathogens most frequently incriminated in OAI will be recognized. Thus, this future more selective sequencing will end up achieving better specificity and improved sensibility and faster identification of suspected pathogens, thereby reducing the overall costs of the investigations (28).

## 7 Liquid biopsy as a non-invasive diagnostic approach

Identifying the pathogen responsible for an infection is essential since this is a precondition to tailoring a definitive antibiotic treatment regarding the therapy type, route, and duration (38, 39). In addition, recognition of the pathogen and its virulence factors may also condition the need and indication for complementary surgical treatment (38). Currently, the pathogen's identification involves carrying out an invasive surgical procedure that we would like to be able to avoid in certain circumstances. Thus, the need to identify the pathogen responsible for an OAI seems to constitute a sufficient element for performing, at least, an arthrocentesis or a bone punction (38).

Liquid biopsy, which involves detecting pathogen DNA in plasma using mNGS, represents a revolutionary approach to diagnosing OAIs caused by *K. kingae*, without invasive procedure. The mNGS method performed in plasma samples can detect not only pathogens circulating in the bloodstream but also those responsible for focal infections (40). In fact, DNA from infected joints, bones, or other musculoskeletal tissues is released into the bloodstream, where it can be detected and identified using mNGS (40). For *K. kingae*, the pathogen's DNA will be released from infected sites as joints, bone or other osteoarticular tissues to the patient's blood where it can be potentially detected and identified with certainty and security (41). A multicenter study recently demonstrated the efficacy of liquid biopsy in diagnosing *K. kingae* spondylodiscitis in infants, where conventional blood cultures failed to identify the pathogen (41). About it, mNGS performed in plasma with a commercially available test, Karius test, Redwood city, CA, USA, allowed the detection of *K. kingae* in 10 infants with spondylodiscitis. Thus, the detection of *K. kingae*'s DNA by liquid biopsy enabled an adapted and adjusted antibiotic treatment in 90% of infants with spondylodiscitis (41).

This method has the potential to eliminate the need for source sample collection via invasive and costly surgical procedures, and this is especially true since most of OAIs caused by K*. kingae* do not require surgical procedures for their treatment.

## 8 Nanotechnology-based diagnostic tools: future perspectives

Nanotechnology-based biosensors have demonstrated increased sensitivity in detecting various analytes, including bacterial species (42–44). The typical biosensors consist generally of immobilized bio-sensitive materials as recognition moieties (a part of a specific molecule) and physical or chemical transducers that translate the recognition information into measurable signals. Commonly employed bio-recognition elements involve antibodies, nucleic acid derivatives, peptides, enzymes, or whole cells (45, 46). The targets of nanoparticle-based biosensors (NPB) for the detection of pathogens can be therefore either specific purified proteins or receptors on the surface of pathogen microorganisms (47), bacterial virulence factors (48, 49), the whole cells (50). To date, a considerable number of biosensors, answering to the name aptamers, have been developed and selected against versatile pathogenic species. Although no biosensors have been developed exclusively for *K. kingae*, the technologies used for other bacterial microorganisms may be adapted to this specific osteoarticular pathogen. Despite these encouraging advances, major challenges remain and revolve mainly around the need to standardize diagnostic methods and to ensure their accessibility in healthcare settings.

## 9 Conclusions

This review examines the roles and utility of traditional and modern diagnosis methods in managing *K. kingae* OAIs in children. While conventional culture methods remain insufficient, advances in molecular techniques, particularly NAAAs and mNGS, have greatly improved pathogen detection. The future of *K. kingae* diagnosis probably lies in the continued development of non-invasive methods such as liquid biopsy and nanotechnology-based sensors, which promise to enhance diagnostic accuracy while minimizing the need for surgical procedure.
